# The Gluten-Free Diet: The Road Ahead

**DOI:** 10.3390/nu17071226

**Published:** 2025-03-31

**Authors:** Carlo Catassi, Fernando Gabriel Chirdo

**Affiliations:** 1 Department of Pediatrics, Polytechnic University of Marche, 60121 Ancona, Italy; 2 Instituto de Estudios Inmunologicos y Fisiopatologicos—IIFP, Departamento de Ciencias Biologicas, Facultad de Ciencias Exactas (UNLP-CONICET), La Plata 1900, Argentina; fchirdo@gmail.com

The gluten-free diet (GFD) is the resolutive and safe treatment of celiac disease (CeD), i.e., one of the commonest, life-long human diseases affecting 1–2% of the general population in most countries [1]. First established about 85 years ago by the Dutch pediatrician William Dicke [2], who certainly deserved the Nobel Prize but could not be nominated due to his premature death in 1962, the GFD has saved thousands of lives and is improving the health and quality of life of millions of people every day. Interestingly, the GFD is one of the few examples in medicine of a treatment that has survived almost unchanged over such a long period of time. Rather, the huge increase in CeD incidence over time, together with the characterization of other common gluten-related disorders like non-celiac gluten sensitivity and wheat allergy, led to an exponential use of the GFD in clinical practice [1]. The papers collected in this Special Issue on “Recent Advances in Gluten-Free Diet and Celiac Disease” address current topics on CeD diagnosis and the GFD, including the psycho-social aspects of this important treatment.

Over time, there has been a substantial and positive evolution in the number, variety, and quality of gluten-free products available on the market, in terms of ingredients, nutritional value, appearance, texture, flavor, and microbial aspects. However, in parallel with the huge spread of the GFD, new issues are emerging that might drive further improvement of gluten-free products in the near future, such as the following:

1. Revision of the current gluten threshold in products labeled as gluten-free: since the year 2007, the Codex Alimentarius recommends that food labeled as “gluten-free” contains a maximum amount of 20 mg per kg (20 parts per million = ppm) of product. The “20 ppm rule” has been adopted in many countries, such as the USA, Canada, some Latino American countries, the UK, and the EU. This limit (so-called gluten threshold) is based on a limited number of available data suggesting that the ingestion of up to 10 mg per day of gluten is not harmful for the vast majority of patients. However, new lines of evidence indicate that the upper limit of gluten tolerance in CeD is not only very low but also consistently variable between patients, a subgroup of them being intolerant even to gluten traces [3]. Furthermore, gluten-free products are also used by patients with wheat allergy, a condition that may be characterized by severe reactions even to a few mg of ingested gluten [4]. In order to protect all the “hypersensitive” patients, a reduction in the gluten threshold in labeled gluten-free products, e.g., from 20 ppm to “no detectable gluten”, could be the way to go in the near future. After all, “gluten-free” means “containing no gluten”.
2. Fortification of gluten-free food: micronutrient deficiencies are not uncommon in CeD, particularly iron deficiency; therefore, fortification needs special consideration. Iron deficiency is very frequent at both CeD diagnosis and follow-up since iron is physiologically adsorbed in the proximal portion of the small intestine, i.e., the site of the celiac lesion [5]. A persisting iron deficiency may suggest incomplete recovery of the small intestinal mucosa after starting treatment with GFD, for instance due to repeated gluten contaminations of the diet. Therefore, supplementation of gluten-free food with iron could be appropriate, and this should take into consideration the bioavailability of the iron supplement. Deficiency of vitamin B12 and folates may also occur in celiac patients, particularly at diagnosis and in patients on treatment with some drugs, e.g., proton pump inhibitors, that can reduce the absorption of vitamin B12. Deficiency of vitamin D is common in celiac patients, both at diagnosis and follow-up; therefore, fortification with this vitamin could be appropriate as well [6].
3. CeD-associated risk of Metabolic Dysfunction-Associated Steatotic Liver Disease (MASLD): in recent years, it has been shown that individuals with treated CeD are more prone to developing MASLD and/or metabolic syndrome than controls, for several reasons including improved nutrient absorption and weight gain on a GFD, increased intake of simple carbohydrates and saturated fats, decreased intake of complex carbohydrates and fibers, CeD-associated dysbiosis, and stress-induced emotional eating leading to weight gain [7]. Supplementation of gluten-free products with specific fibers with favorable effects on lipid metabolism and insulin resistance, such as inulin [8] and beta-glucans [9], may help prevent and/or treat this emerging comorbidity.
4. Treatment of CeD-associated dysbiosis: dysbiosis, i.e., a qualitative and/or quantitative alteration of the intestinal microbiota, is a hallmark of CeD, persists across various disease stages, and is only partially corrected by treatment with the GFD [10]. In untreated CeD, the microbiota is markedly unbalanced with a reduction in beneficial microbes like Lactobacillus and Bifidobacterium and an increase in pathogenic bacteria such as Bacteroides and E. coli. The increase in pathogenic bacteria could play a crucial role in fostering pro-inflammatory responses against gluten [11]. Supplementation with live bacteria (probiotics) could modulate the composition and functions of the microbiota, in order to mitigate persisting gut inflammation in CeD patients treated with the GFD. Prebiotics, i.e., substrates that are selectively utilized by host microorganisms conferring a health benefit, are conceptually more suitable for supplementation of industrially prepared food undergoing cooking processes under high temperatures (e.g., bread, cookies and so forth), rather than the addition of viable microorganisms. The favorable effects of some prebiotic compounds, such as oligofructose-enriched inulin, on CeD-induced gut inflammation have been experimentally confirmed [12].

“Food as a Medicine” is a concept that perfectly fits for the GFD. Doctors and dietitians specializing in CeD play a critical role in the education and maintenance of the GFD for patients with CeD [13]. As with many other medical treatments, the GFD is evolving toward a more personalized approach, in terms of efficacy, safety, and nutritional value. Although alternative treatments to the GFD, such as enzymes, drugs, or even vaccines, are currently under the scrutiny of research, it is not difficult to predict that an optimized GFD will remain the elective treatment of CeD and other gluten-related disorders for many years to come.

## Abbreviations

The following abbreviations are used in this manuscript:

GFD	gluten-free diet
CeD	celiac disease
ppm	parts per million
MASLD	Metabolic Dysfunction-Associated Steatotic Liver Disease
